# Navigating recovery in childhood OCD: a qualitative analysis of barriers and facilitators

**DOI:** 10.1186/s13034-024-00851-8

**Published:** 2024-12-18

**Authors:** Lakshmi Sravanti, Rajendra Kiragasur Madegowda, Arul Jayendra Pradeep Velusamy, John Vijay Sagar Kommu, Satish Chandra Girimaji, Shekhar Seshadri

**Affiliations:** 1 Department of Child and Adolescent Psychiatry, National Institute of Mental Health and Neurosciences (NIMHANS), Bengaluru, 560029 India; 2 Specialty Registrar in Child & Adolescent Psychiatry, Oxfordshire CAMHS NDC Pathway Team, Oxford, UK

**Keywords:** Recovery, Childhood obsessive-compulsive disorder, Barriers, Facilitators

## Abstract

**Objective:**

The objective is to examine barriers and facilitators to recovery in children and adolescents with obsessive-compulsive disorder (OCD) using a qualitative approach.

**Methods:**

Ten semi-structured interviews were conducted, audio-recorded, and analyzed using thematic analysis. Findings were validated through investigator triangulation, peer validation and member check.

**Results:**

Barriers to recovery were internal—lack of awareness; poor motivation to seek treatment; and perceived stigma, or external—poor parental support; parental anxiety; inadequate awareness in schools; social misconceptions about illness; myths about medication; and frustrations in treatment processes. Facilitators were internal—will and determination; self-discipline; keeping calm; sense of purpose, and external—general awareness; parental support; peer support; and good therapeutic engagement.

**Conclusions:**

To the best of our knowledge, this is the first study to explore barriers and facilitators to recovery in-depth in pediatric OCD. Findings underscore the importance of tailored interventions, robust support networks, and cultural sensitivity for successful recovery outcomes.

**Supplementary Information:**

The online version contains supplementary material available at 10.1186/s13034-024-00851-8.

## Introduction

Obsessive-Compulsive Disorder (OCD) manifests as a chronic illness necessitating prolonged maintenance treatment to mitigate a high risk of relapse. Left untreated, OCD tends to follow a chronic course, leading to substantial functional impairment and an increased susceptibility to other psychiatric disorders in adulthood [1, 2]. Despite advancements in treatment modalities, a significant proportion, one in ten individuals with OCD, remains severely affected [3]. A meta-analysis of 16 studies was conducted on outcome data, the samples of which had a follow-up duration ranging from one to 15.6 years. Pooled mean persistence rates were 41% and 60% for full OCD and full or subsyndromal OCD respectively [4]. Reddy et al. followed 58 drug-naïve children in India for a mean duration of 5 years and reported that 48% were in true remission (symptoms remitted and not on treatment) and 21% had clinical OCD at follow-up [5].

When OCD manifests in childhood, it disrupts normal development and heightens the likelihood of anxiety disorders in adulthood [2]. Factors such as the presence of a triggering event, episodic course, and fair socio-occupational adjustment may contribute to a more benign disease course. Predictors of remission include an older age at onset, outpatient status, and a shorter duration of illness, as highlighted in the work of Sadock and Sadock [6]. Access to treatment significantly influences the course of OCD. Research in adult patients with OCD identified various factors affecting access including the cost of treatment, lack of insurance coverage [7], lack of transportation, long waitlists, and a shortage of trained providers/therapists [8]. Additionally, parents of children with OCD reported difficulties navigating the mental health system, often spending years searching for an exposure therapist, with search sometimes hindered by fluctuating symptoms [8]. These insights underscore the intricate interplay of various factors influencing the trajectory and outcomes of childhood-onset OCD.

In the Indian context, the family plays a central role in a child’s development, with strong intergenerational ties and collective decision-making, particularly in health matters. Cultural norms prioritize respect for elders, obedience, and academic success, which leads children to place significant importance on how their parents respond to mental health concerns [9]. Religious beliefs also hold weight, with many seeking traditional healing methods, such as faith healers or religious shrines, alongside or instead of formal treatment [10]. Furthermore, societal views on mental illness in India are often stigmatized, with mental health issues sometimes being linked to shame or moral failings [11]. This stigma can delay treatment and discourage open discussion, adding additional challenges to accessing care. Socioeconomic and geographic factors, such as living in rural areas or lower-income backgrounds, can further limit access to mental health services, contributing to disparities in care. Understanding these cultural, familial and socioeconomic factors is crucial to interpreting the experiences of children with OCD in India.

Quantitative research predominates in the study of OCD among children, contrasting with a scarcity of qualitative data. To our knowledge, only two qualitative studies have delved into the lived experiences of children and adolescents with OCD [12, 13]—one of which is the first part of this study that comprehensively analyzed the subjective experiences of children and adolescents living with OCD [13]. The majority of current findings stem from research involving adult populations. Table 1 summarizes the relevant findings of two studies that explored the domain of recovery. Given the developmental differences, it will be worthwhile studying the barriers and facilitators to recovery in children with OCD. The current paper addresses this felt need to study the first-hand accounts of children and adolescents suffering from OCD with a focus on the process of recovery.

**Table 1:** Qualitative research exploring the barriers and facilitators to recovery in OCD

Authors & Place	Phenomenon	Sample	Method	Key findings
1. Robinson, Rose and Salkovskis [14], (UK)	Enablers & barriers	*n* = 17	Thematic analysis	Barriers were stigma; ‘internal/cognitive’ factors (not knowing what their problem was); factors related to treatment or general practitioner; and fear of criminalisation. Positive enablers were support to seek help; information and personal accounts of OCD in the media; and confidence in general practitioner. Negative enablers identified were crisis point; for some it was feeling driven to seek treatment because of the nature of content of the thoughts, i.e. seeking help to prevent the ‘harm’ they feared they were capable of doing (whose intrusive thoughts were about harming children).
2. Pedley et al., [15](UK)	Family members’ perceptions	*n* = 14 (dyads); 16 years or older	Thematic analysis	Main themes—OCD viewed as arising from non-modifiable endogenous factors (personal characteristics); lack of distinction between behaviours performed for happiness and problematic behaviours; OCD on continuum with sub-clinical symptoms; labelling of subclinical symptoms by public led to frustration of families; and pessimism regarding recovery.

## Methodology

### Study design

A qualitative study using in-depth, semi-structured interviews was conducted at the National Institute of Mental Health and Neuro Sciences (NIMHANS). Children and adolescents diagnosed with obsessive-compulsive disorder were recruited through purposive sampling to conduct interviews in English. In qualitative research, homogeneity within the sample enhances understanding of the studied phenomenon. In this study, participants were selected based on having OCD for at least six months and being in remission at intake. Remission was defined, following Mataix-Cols et al. [16], as either not meeting OCD criteria for one week in structured interviews or scoring ≤ 12 on the Children’s Yale-Brown Obsessive Compulsive Scale (CY-BOCS) and 1–2 on the Clinical Global Impression Scale-Severity (CGI-S) for at least one week. This ensured all participants were in remission during the interviews, allowing them to fully reflect on barriers and facilitators in their recovery.

Two of the twelve participants who provided assent and had family consent withdrew from the study due to difficulty expressing themselves fluently in English during the detailed interview. Face-to-face interviews were conducted in the out-patient and in-patient settings of the department of child and adolescent psychiatry, NIMHANS till the saturation of themes occurred. Only the participant and the interviewer were present in the room during the interview. Theme saturation was reached with the tenth participant. The interviews were audio-recorded, conducted in one or two sessions, with no interviews being repeated.

### Research questions

Central research question: What are the subjective experiences of recovery in children with obsessive-compulsive disorder?

Issue sub-question: What does recovery signify?

Procedural sub-questions:

1. What is helping in becoming alright?
2. What is coming in the way of becoming completely alright?

### Procedure

The interviews were conducted by a qualified female psychiatrist [LS] with a masters in psychiatry (MD) and extensive experience in qualitative data collection and analysis, particularly in mental health populations, utilizing structured diagnostic interviews and scales to confirm clinical diagnoses and assess severity.

The researcher met with participants and their families prior to the study to explain its purpose and procedures, building rapport to ensure a comfortable interview environment. Participants were informed about the researcher’s expertise in child and adolescent mental health and the goal of understanding the lived experiences of children with OCD to improve therapeutic approaches. The researcher disclosed a professional interest in child mental health and OCD, acknowledged potential biases such as the belief in early intervention, and minimized these biases through reflective practices and peer validation.

The qualitative data was collected using an in-depth, semi-structured interview guide that was informed by existing literature. The preliminary version was pilot-tested with two children to make necessary modifications, and the final version was validated by a peer researcher (AJPV) and two senior researchers (JVSK, SCG) experienced in qualitative methods. The technique of funnelling was employed in constructing the guide, allowing participants to first express general views before addressing specific issues [17]. Developing the interview schedule was a reflective process, mindful of the impact on participants’ lives [18]. Prompts were used to enrich responses, especially when participants had difficulty understanding questions or elaborating on their answers [17].

The questions were open-ended, aiming for descriptive responses without right or wrong answers. Participants were allowed to lead the conversation, ensuring all specific areas were covered while also providing space for any additional relevant ideas. Interviews were audio-recorded to prevent data loss and recall bias, capturing responses exactly as narrated. Audio recordings also included the researcher’s responses and allowed for pausing as needed, aiding in better understanding [19]. Participant behavioural observation is an important concept in qualitative research methodologies [20]. Therefore, notes of these observations were made after the interview. These notes helped to accurately recall the context and meaning of interview. Observations made in the context of specific responses helped us to understand better the emotional underpinnings of the subjective perceptions of our participants.

### Analysis

For the first part of the study, we utilized interpretative phenomenological analysis (IPA), where the primary goal was to explore the lived experiences of children with obsessive-compulsive disorder (OCD) and, to that end, IPA was selected for its focus on understanding participants’ personal, subjective experiences in depth [13]. For the secondary objective, which focused specifically on identifying the barriers and facilitators to recovery, we opted for thematic analysis. Thematic analysis is highly flexible and well-suited for systematically analyzing qualitative data across different contexts and experiences, making it ideal for a structured exploration of patterns within the data. Unlike IPA, which is idiographic and focuses on individual experiences, thematic analysis allowed us to categorize common themes across participants, highlighting shared elements in their recovery journeys while also capturing individual nuances. This approach enables to gain a broader perspective on the factors influencing recovery, with a focus on both the barriers and the facilitators children encounter. Thematic analysis also offers the benefit of adaptability, allowing us to analyze our data without being tied to a rigid theoretical framework, thereby enabling a more data-driven exploration. This flexibility is valuable when addressing specific research questions, like ours, which aim to reveal distinct yet interrelated dimensions of recovery.

The combination of IPA and thematic analysis was strategically selected for the overall study to provide a robust understanding of the subjective experiences of OCD while also identifying tangible factors that impact the recovery process, thereby enhancing the study’s relevance to clinical practice and potential therapeutic interventions.

Thematic analysis, as described by Braun and Clarke [21], involves identifying, analyzing, and reporting patterns (themes) within data. All the audio-recorded interviews were transcribed verbatim (LS). The analysis was an active, researcher-driven process, following the guidelines provided by Braun and Clarke [21]. The primary researcher (LS) familiarized themselves with the data by reading the transcripts multiple times, making initial notes on potential codes. These codes were then systematically developed and reviewed, ensuring they captured the essence of the data.

The codes were organized into broader themes, which were then reviewed and refined to ensure they accurately represented the data. This process involved an iterative cycle of revisiting the data, refining the themes, and ensuring they were coherent and distinctive. The final list of major themes and sub-themes was compiled to provide a comprehensive understanding of the participants’ experiences.

The findings were validated through investigator triangulation, peer validation ensuring rigor in the study by including agreed-upon themes and sub-themes in the final report. The transcripts were not shared with the participants for their comments, however, feedback was sought on the final findings through the process of member checks to ensure interpretive validity [22]. Our findings were presented to the participants in individual sessions in their follow-up visits. The themes and sub-themes upon which there was agreement were included in the final report and where consensus could not be reached were discarded. The study is presented in accordance with COREQ guidelines for reporting qualitative research, and the checklist is submitted as supplementary material.

### Ethical considerations

Permission to conduct the research was granted by the Institutional Ethics Committee of the National Institute of Mental Health and Neurosciences (NIMHANS). The study’s objectives and procedures were thoroughly explained to both the children and their parents, with written and verbal explanations provided. Prior to participation, all participants and their parents provided written informed assent and consent, respectively. Measures were taken to ensure anonymity and confidentiality throughout the study.

The recruitment process and study methodology are comprehensively elucidated in the primary manuscript focusing on the lived experiences of children and adolescents with obsessive-compulsive disorder [13].

## Results

### Sociodemographic and clinical profile

The participants, aged 10 to 17 years, included four girls and six boys, with eight attending regular school, one training in pre-vocational skills, and one pursuing open schooling; the age at onset of illness ranged from 9.5 to 13 years (mean 11, SD 1.2), and the age at diagnosis ranged from 10 to 14.5 years (mean 12.4, SD 1.9). All were from an urban background, four participants were from upper socioeconomic status, four from upper-middle, and two from lower-middle. Eight participants were Hindu, and two were Christian. The clinical profile is detailed in Table 2. At the peak of illness, family accommodation was noted in all participants, and all but three (P2, P6, P9) demonstrated insight into the condition. All received combined pharmacotherapy and cognitive behaviour therapy (CBT), except one (P4) who received only CBT. Six participants (P2, P3, P6, P7, P9, P10) required in-patient treatment during their course of care.

**Table 2:** Clinical profile of the sample

Participant	Age at onset (years)	Age at diagnosis (years)	Duration of illness (months)	Duration of remission (weeks)	CY-BOCS (pre-treatment)	CY-BOCS (at recruitment)	CGI-S	Major symptom dimension
P1	10	10	10	12	24	4	1	Sexual/ Religious
P2	11	14	46	18	30	7	2	Aggression, Contamination, Symmetry
P3	11	11	10	24	15	0	1	Contamination
P4	13	14.5	26	26	21	2	1	Contamination
P5	12	12.5	23	28	22	1	1	Aggression
P6	10	10.5	30	16	37	4	2	Contamination, Symmetry
P7	11	15	52	12	21	3	2	Contamination, Symmetry, Aggression
P8	10	11	30	22	23	0	1	Contamination, Symmetry
P9	9.5	11	19	12	28	4	1	Contamination, Symmetry
P10	12.5	14.5	54	27	24	2	1	Aggression, Sexual/ Religious

### Qualitative interview duration

The interviews ranged from a minimum of 1 h 4 min to a maximum of 2 h 47 min. Average duration of interviews was 1 h 49 min. The number of sessions and total duration per participant is summarized in the table underneath (Table 3).

**Table 3:** Number of sessions and duration of interviews

Serial No.	Participant	Number of sessions	Total duration of interview (min)
1.	P1	2	128
2.	P2	2	167
3.	P3	2	105
4.	P4	1	93
5.	P5	1	72
6.	P6	2	110
7.	P7	2	130
8.	P8	1	72
9.	P9	1	64
10.	P10	1	149

### Results of thematic analysis

Initially the major theme of ‘recovery as a process’ will be elaborated. Further, the barriers and facilitators to recovery derived by thematic analysis are detailed, each sub-theme validated with at least three significant statements.

#### Major theme: Recovery as a process

Generally, recovery was perceived as a ‘process’ by the participants, whether fluctuating in course or steady improvement (Table 2).

##### Sub-theme

Ups and downs.

P7: “Moving forward, It may not be an easy ride, there are going to be ups and downs but it will be entertaining.” (16 year old girl).

P8: “It has been a mix of emotions through the journey. […] it is going to be difficult from here on. It may not go smoothly. […] OCD may affect course of my journey but I should be prepared for it.” (12 year old boy).

P10: “I am better prepared for the future. […] it is going to be ups and downs but I will be able to overcome it better because I experienced difficulties already, so I will be able to face better.” (17 year old girl).

##### Sub-theme

Slow and steady.

P1: “First it was like very difficult. Slowly, it became easier. Now, it is much easier than before. Gradually, it will further reduce and go away.” (10 year old boy).

P4: “It is like a continuous process. I just want to keep developing. I want to keep improving with regard to OCD, slowly and steadily.” (16 year old girl).

P9: “I want to become something. I want to achieve something. It may take a lot of time but to get there I have to start working on a lot of things. Gradually over time I am sure I will get complete control over myself and my behaviours.” (11 year old boy).

### Barriers and facilitators to recovery

The personal and socio-cultural influences on the pathways to recovery forming the barriers and facilitators are presented below. ‘Internal’ and ‘external’ are the two major themes identified for both barriers and facilitators to recovery in childhood OCD. The identified sub-themes are systematically organized in a logical sequence, progressing from awareness to help-seeking, through the treatment process, and finally to post-treatment wherever possible.

### Barriers to recovery

The three sub-themes classified as ‘internal’ and the six sub-themes categorized as ‘external’ are illustrated below with three excerpts from the interviews for each sub-theme.

#### Major theme: Internal

##### Sub-theme 1

Lack of awareness.

P2: “I didn’t know this could be a mental illness. I had no clue was going on, so I didn’t talk to anyone about it.” (15 year old boy).

P4: “No one knew what was going on. It took me 3 years to seek treatment.” (15 year old girl).

P7: “Had I known that the kind of thoughts I had and my behaviours were a disease, I would have sought help much earlier.” (16 year old girl).

##### Sub-theme 2

Poor motivation to seek treatment.

P1: “I didn’t want to go to the doctor as I was scared. It was very difficult for my parents to bring me here.” (10 year old boy).

P3: “My own laziness comes in way of following the strategies learnt in sessions. I am trying to overcome it.” (11 year old boy).

P9: “I was suffering but going to a doctor was not something I liked to do at all.” (11 year old boy).

##### Sub-theme 3

Perceived stigma.

P1: “I was not willing to meet doctor. I was not ready to talk about the thoughts I was getting because they were really dirty thoughts. I was worried what doctors would think of me.” (10 year old boy).

P8: “When I was asked by my friend about tablets I took and I couldn’t answer, I felt bad. At times, I didn’t want to take medicines to avoid embarrassment.” (12 year old boy).

P10: “When I tried to talk about my problem with my mother. She reacted by saying ‘Ok, shall I ask your father to take you to mental hospital’. That put me off from saying anything. I was scared if they would label me as being ‘mad’ or ‘mental’.” (17 year old girl).

#### Major theme: External

##### Sub-theme 1

Poor parental support.

P1: “In the beginning my parents used to say, ‘why are you getting these thoughts, you should stop, you should control your thoughts’. But they failed to understand that it was not under my control. I would feel very guilty and blame myself for the thoughts.” (10 year old boy).

P7: “When I first realized that my behaviour was a problem and my parents refused to accept the condition, I felt that ‘my parents are not with me’. There is a mismatch in my views and that of my parents’– so this thing is always a BIG deal for me.” (16 year old girl).

P8: “I don’t think my father understood me or what I was going through. I didn’t know who else to go to.” (12 year old boy).

##### Sub-theme 2

Parental anxiety.

P6: “My parents are worried that my OCD might come back again. I don’t want them to worry about that.” (12 year old girl).

P8: “I felt sad when my father asked doctor the details of my problem. He appeared worried. When I see my father like that, I worry more.” (12 year old boy).

P10: “When I was ill, I wanted my mother to help me but my mother used to cry. She could have said, ‘Ok, come let’s go out for a walk’, ‘don’t cry’ and things like that would have helped me feel better.” (17 year old girl).

##### Sub-theme 3

Inadequate awareness in schools.

P2: “At school, I could not focus on classwork. I used to go blank… teachers shouted at me and complained to my parents saying I was not following their instructions.” (15 year old boy).

P3: “Because of the OC symptoms many teachers thought I was a dumb student. I couldn’t even say a word about how I was feeling’.” (11 year old boy).

P10: “In my 8th grade when I didn’t go to school, there were some rumours that I was possessed by a devil. Same thing happened when I was in 10th standard also. It made me feel sad.” (17 year old girl).

##### Sub-theme 4

Social misconceptions about illness.

P2: “People thought I did drugs. Some said it was because I played ‘Blue Whale’ game that I became like that. I wonder why they said things like that.” (15 year old boy).

P7: “Once at a family function, my relatives noticed my repetitive behaviours and frowned at me. My aunt said I had got this problem for my wrong-doings in my past life. Some others said I was cursed and I won’t be happy in the future.” (16 year old girl).

P10: “My aunt called me and said it was the effect of evil eye or black magic and that I should seek help of faith-healers and perform some rituals to get rid of it. My neighbours stopped coming to my house. I overheard some people talking about me and calling me ‘mental’, an ‘idiot’ and saying that no one should talk to me.” (17 year old girl).

##### Sub-theme 5

Myths about medication.

P2: “My friends and relatives ask me, ‘Why you are taking tablets? It is not good. They are harmful.’ I worry about what will happen as I have been taking medicines for a long while.” (15 year old boy).

P8: “My family members are worried that it may have side-effects for having used for so long. They say that I should put in effort to control my thoughts and behaviours.” (12 year old boy).

P10: “I read online that medicines are harmful. They may cause problems to kidney. I have irregular periods, I don’t know if it is because of this medicine. My father too expresses his concern and wishes that I learn to control my problem without relying on medicines.” (17 year old girl).

##### Sub-theme 6

Frustrations in treatment process.

P5: “I must have seen at least 10 doctors. We were going to different doctors and they were prescribing skin creams.” (14 year old boy).

P9: “The first doctor was a general physician who referred me to a psychiatrist. Then we consulted a number of doctors. Different medications were prescribed for me [..]. My parents were going to and fro between allopathic doctors, homeopathic and ayurvedic doctors. [..] I was being taken to temples as well. I had no idea what was happening and I was suffering all along.” (11 year old boy).

P10: “I visited a dozen doctors. I met different people from neurologists to even faith-healers. I had some very scary experiences. No one understood what was going on with me.” (17 year old girl).

### Facilitators to recovery

The four sub-themes classified as ‘internal’ and the four sub-themes categorized as ‘external’ are illustrated below with three excerpts from the interviews for each sub-theme.

#### Major theme: Internal

##### Sub-theme 1

Will and determination.

P7: “I have set a lot of goals for myself. I think OCD can affect the process but I can affect OCD too.” (16 year old girl).

P8: “It has been a mix of emotions through the journey. It is going to be difficult from here on. It may not go smoothly but I should be prepared for it.” (12 year old boy).

P10: “I have experienced a lot of suffering. But, I am better prepared for future now. It is going to be ups and downs but I will be able to overcome it.” (17 year old girl).

##### Sub-theme 2

Self-discipline.

P2: “I should not repeat my action and I should just let go. By regular practice, OCD can come down! (15 year old boy).

P5: “Following a good routine that includes taking tablets, practicing mindfulness will go a long way in making one feel better. I am also keen on learning more exercises and techniques that I can practice at home that will help the course of this journey.” (14 year old boy).

P10: “If I exercise self-discipline, I may feel more confident to overcome those situations where OCD is affecting me.” (17 year old girl).

##### Sub-theme 3

Keeping calm.

P5: “I want to stay calm. Doing mindfulness has really helped me feel better.” (14 year old boy).

P6: “Practising relaxation or doing activities like reading books and listening to music have helped me better able to handle day-to-day problems.” (12 year old girl).

P7: “I want to be at peace with myself. OCD definitely disturbs that equilibrium but putting effort to stay calm and relaxed in every situation helps in fighting against OCD itself.” (16 year old girl).

##### Sub-theme 4

Sense of purpose.

P3: “I know OCD is just one bit in the bigger picture called life. but yeah right now it is an important piece of the puzzle.” (11 year old boy).

P6: “I set myself some goals, but OCD may come in way of doing certain things that I wish to do. So, if I try to get rid of it then maybe I would be able to do all that also.” (12 year old girl).

P10: “I envision my dream… in fact every night I remind myself of what goals I have set for myself in life and imagine achieving them. This helps me bring my focus back to working on daily hassles because of OCD.” (17 year old girl).

#### Major theme: External

##### Sub-theme 1

General awareness.

P1: “My aunt told my mom that it could probably be OCD and I would benefit from medications. That is why we came here for consultation.” (10 year old boy).

P5: “My aunt brought me to hospital the first time. […] if people know about OCD, they will come to know early when illness starts.” (14 year old boy).

P10: “A distant relative of ours, told my parents to consult a neurologist they knew. We were directed to a psychiatrist from there and subsequently got the right kind of help.” (17 year old girl).

##### Sub-theme 2

Parental support.

P5: “My mother has been my biggest source of support. On noticing my behaviours, she took me for help in the initial period itself.” (14 year old boy).

P6: “My parents have always been there to support me and they kind of remind me like how to tackle by saying “remember what they taught you”—so that helps me.” (12 year old girl).

P9: “My parents have helped me reduce my compulsive behaviours. My life was derailed, it has been a very difficult journey. My father’s support mattered the most.” (11 year old boy).

##### Sub-theme 3

Peer support.

P4: “A friend of mine has anxiety and she was doing CBT through an app. She said that therapy really helps and encouraged me to go.” (15 year old girl).

P7: “Going forward, I think I want to take my friends’ help. At times, they forced me to try the street food, which I always avoid. It helped me facing my fears to some extent.” (16 year old girl).

P9: “I wish to have true friends with whom I can share everything—from things related to studies and games to problems I face and difficulties I go through.” (11 year old boy).

##### Sub-theme 4

Good therapeutic engagement.

P5: “If doctors spend more time trying to understand the situation, one will feel comfortable to talk about all the issues. [.].having a good relationship with doctor is very important!” (14 year old boy).

P6: “Maybe I would say that talking to a professional you trust would reduce stress and make one feel better.” (12 year old girl).

P10: “In therapy, I could express myself and my feelings freely. Having a professional or a therapist as part of the support system helps immensely in the recovery process.” (17 year old girl).

## Discussion

### An analysis of the barriers to recovery

The delay in recognizing the problem often stems from a lack of awareness, leading to postponement in seeking assistance and consequently affecting the trajectory of illness and recovery. Instances reveal children’s reluctance to pursue treatment despite recognizing their issues, highlighting a concerning lack of motivation. Furthermore, stigma plays a pivotal role in hindering help-seeking behaviors, exacerbating its detrimental impact when it impedes treatment adherence. Notably, stigma may also arise from the nature of one’s thoughts, inhibiting open discussions with therapists. In the Indian context, stigma surrounding mental illness is reinforced by hierarchical family structures, where parental authority shapes children’s experiences. This often leads to reluctance in discussing mental health, as deviation from societal norms can evoke shame and hinder treatment-seeking [23]. In our study, children express feelings of invalidation, leading to emotional distress, compounded by a sense of guilt and self-blame originating from expectations of self-control over their thoughts. This emotional burden impairs their coping abilities. The home environment, primarily shaped by parental reactions, significantly influences how children manage challenges. Narratives underscore the detrimental effect of parental anxiety on children’s worries. School environments also play a crucial role in a child’s development, yet a lack of understanding about their illness within this context exacerbates their struggle to return to normalcy. Social beliefs about illness causation, often rooted in culture and faith rather than medical understanding, further complicate matters [10]. Concerns about medication arise from unfounded beliefs rather than scientific knowledge, adding to pressure for self-control over reliance on medication, which can hinder therapy progress. Perceived stigma and delays in seeking help also hinder recovery, with various treatment pathways and therapist-related factors impacting a child’s journey through illness [11].

### An analysis of the facilitators to recovery

Children emphasize the importance of dedicated determination and a step-by-step approach to combat OCD. Some statements highlight self-discipline ranging from restraining one’s actions and emotions to establishing a healthy routine to overcome the disorder. They recognize that maintaining a sense of calmness and relaxation aids in managing OCD-related challenges and improves overall functioning. Additionally, they find purpose in striving for a better future, prioritizing the resolution of OCD issues in their journey forward. In all the cases, it was a family member who noticed problematic behaviors and guided the family toward seeking appropriate assistance for the condition. This underscores that when there is widespread awareness about the condition, it facilitates recognition and help-seeking, even if parents are unable to identify the behaviors as problematic. These accounts underscore the crucial role of parental support throughout various stages of the illness. Parents provide a foundation for children to lean on in their battle with OCD and throughout the subsequent recovery journey. Some excerpts indicate the significance of peer support in the path to recovery, with children noting how it positively influenced their feelings or expressed a desire for supportive friends to discuss OCD-related challenges. These narratives stress the importance of a strong therapeutic relationship during treatment and recovery. It’s noteworthy that children recognize the value of rapport in the therapy process.

### Comparing study findings with relevant literature

The overall themes of ‘ups and downs’ and ‘slow and steady’ observed in this study align with the episodic and chronic courses of OCD described in larger quantitative studies involving children with OCD [24, 25]. Farrell et al. [26] discuss the treatment and quality gaps in OCD care for children. Our findings align with this perspective, as the narratives from our participants identify significant barriers—such as stigma, low treatment motivation, and lack of parental support—that continue to limit access to evidence-based treatments like CBT with exposure and response prevention (CBT-ERP). The treatment gap refers to the large number of children who do not receive mental health services for OCD, while the quality gap pertains to those who access services but do not receive evidence-based treatments such as CBT-ERP.

Barriers to seeking treatment also serve as barriers to recovery, while facilitators in accessing treatment indirectly support the recovery process. This study’s findings on barriers and enablers align closely with those identified by Robinson, Rose, and Salkovskis in their study of adults with OCD [14]. Barriers such as “lack of awareness,” “low motivation to seek treatment,” “perceived stigma,” and “frustrations in the treatment process” are akin to themes like “not knowing what it was,” “internal delays,” “stigma,” and “GP/treatment-related issues” in the adult sample. While Robinson et al. identified “fear of criminalization” as an additional barrier for adults with OCD, our study identified unique external factors influencing children and adolescents, including “poor parental support,” “parental anxiety,” “inadequate school awareness,” “social misconceptions about the illness,” and “myths about medication.” These findings suggest that recovery processes in children and adolescents are influenced by a broader array of external factors, particularly those related to family, school, and community contexts.

Regarding facilitators, our findings on “parental support” and “peer support” align with Robinson et al.‘s theme of “support or encouragement to seek treatment.” Other themes such as “willpower,” “general awareness,” and “effective therapeutic engagement” align with “driven by the nature of thoughts,” “media/information,” and “confidence in healthcare providers,” respectively. Notably, Robinson et al.‘s facilitator theme “crisis or crunch point,” relating to the initial motivation to seek treatment, was absent in this study as our focus was on factors influencing recovery rather than initial treatment-seeking. Consistent with Kohler, Coetzee, and Lochner’s work on “coping strategies” and “perceived social support” under the theme “managing disruptions to daily life,” our findings indicate that social support is a pivotal external factor, with the role of parental and peer support emerging as particularly influential in the recovery process [27]. Additionally, Frank et al. [8] highlighted the complex emotional experiences of parents seeking treatment for their children, including feelings of isolation, hopelessness, guilt, and worry. In line with these findings, our study identified parental anxiety as a substantial barrier to recovery, as reported by the children themselves.

### Clinical implications of themes derived

The clinical implications of the themes derived from the study suggest several key considerations for mental health practitioners:

- Recognize the variability in recovery processes and adopt flexible, individualized treatment approaches tailored to the needs of both the patient and their family. For example, this may include addressing parental anxiety and dispelling myths surrounding medication use.
- Recognize the critical role of external facilitators, such as parental and peer support, in the recovery process. Clinicians should actively involve family members and peers in treatment planning by providing psychoeducation and fostering their active participation in the management plan to support the patient’s recovery journey.
- Consider cultural factors when designing interventions to address social misconceptions that may lead individuals to seek magico-religious practices, potentially delaying timely identification and management. Awareness programs targeting both community and school settings can be effective in this regard. However, it is essential for clinicians to remain sensitive to cultural beliefs and practices, using psychoeducation to engage families and foster a strong therapeutic alliance.
- Regularly monitor patients’ progress, adjusting treatment strategies as necessary to align with their evolving needs and experiences. Ongoing assessment and collaboration with patients enable the identification of potential barriers to recovery, allowing for tailored interventions that optimize therapeutic outcomes.

### Strengths and limitations

To the best of our knowledge this is the first study to explore the barriers and facilitators along the journey of recovery in children and adolescents with OCD done using interpretative phenomenological analysis. Extensive interviews were conducted with all subjects, yielding a wealth of data which was then meticulously analyzed to produce the findings. It has the limitations of a qualitative study such as a lack of generalizability and recall bias. The interviews were conducted in English to ensure consistency and minimize variability in interpretation or translation. However, excluding participants from non-English-speaking backgrounds may have limited the diversity of perspectives captured and introduced the possibility of selection bias, potentially underrepresenting families with less exposure to English education. Factors such as rural versus urban living and socioeconomic status did not emerge prominently in participants’ narratives, likely due to the homogenous urban sample, which limited variation in contextual factors. Additionally, the focus of the participants’ narratives was primarily on personal treatment experiences and cultural beliefs, rather than broader environmental or socioeconomic influences.

### Recommendations for future research

Future longitudinal studies can further explore the dynamic nature of recovery processes, including the fluctuations and pacing described by participants. This could provide deeper insights into the trajectories of recovery over time. The identified internal and external facilitators of recovery can be used as a basis for developing and testing targeted interventions aimed at enhancing recovery outcomes for individuals facing mental health challenges. It would be worthwhile, to investigate the role of community and school-based interventions in addressing external barriers to recovery, such as inadequate awareness in schools and social misconceptions about illness. Collaborative efforts involving schools, mental health professionals, and community organizations may be beneficial.

## Supplementary Information

Supplementary material 1

## Data Availability

No datasets were generated or analysed during the current study.

## Abbreviations

CBT: Cognitive behaviour therapy
MINI KID: Mini International Neuropsychiatric Interview for Children and Adolescents
NIMHANS: National Institute of Mental Health and Neurosciences
OCD: Obsessive-compulsive disorder
